# Urothelial Carcinoma of the Bladder in Pediatric Patient: Four Case Series and Review of the Literature

**DOI:** 10.4274/balkanmedj.2017.1292

**Published:** 2018-05-29

**Authors:** Murat Uçar, Metin Demirkaya, Berna Aytaç Vuruşkan, Emin Balkan, Nizamettin Kılıç

**Affiliations:** 1 Division of Pediatric Urology, Department of Pediatric Surgery, Uludağ University School of Medicine, Bursa, Turkey; 2 Division of Pediatric Oncology, Department of Pediatrics, Uludağ University School of Medicine, Bursa, Turkey; 3 Department of Pathology, Uludağ University School of Medicine, Bursa, Turkey

**Keywords:** Bladder, children, transitional cell carcinoma, treatment

## Abstract

**Background:**

Urothelial carcinoma of the bladder is a rare condition in children, and most cases in this age group are noninvasive and low-grade. However, no follow-up protocol has been defined for this patient group. The objective of this study was to draw attention to bladder tumors in children and focus on the current recommendations for postoperative follow-up along with a case study of four patients.

**Case Report:**

Four patients aged <18 years with urothelial carcinoma who were treated in our clinics between 2001 and 2015 were retrospectively evaluated. The results were compared with those of published pediatric case series in the literature. No abnormalities were found in the patients’ physical examinations and laboratory analyses, except hematuria (microscopic or macroscopic). Ultrasonography was used in all the patients to detect lesions in the bladder. Surgical resections were performed endoscopically, except in one patient. Histopathological evaluations revealed low-grade superficial urothelial carcinoma. No recurrence or complication was observed for all patients.

**Conclusion:**

Although rarely encountered during childhood, urothelial carcinoma should be considered as a differential diagnosis in pediatric patients with hematuria.

Carcinoma of the bladder is uncommon in children. Most bladder tumors are of mesodermal origin; epithelial carcinomas are less common and extremely rare in children. Compared with adult patients, urothelial carcinomas (UCs) diagnosed in children are solitary, noninvasive, and low-grade tumors with minimal potential for recurrence and progression (1,2,3). Here, we report our experience of four pediatric cases of urothelial bladder tumors, with the aim of focusing on treatment and follow-up protocols.

## CASE PRESENTATION

**Figure 1:**
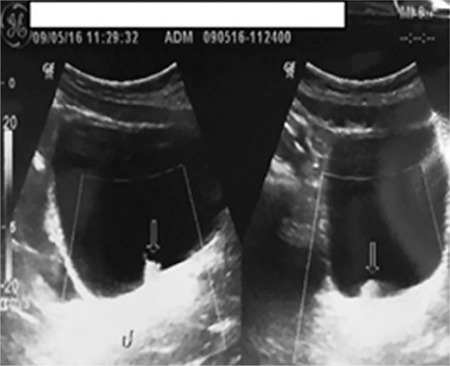
Preoperative ultrasound showing patient’s 4 bladder wall mass.

**Figure 2:**
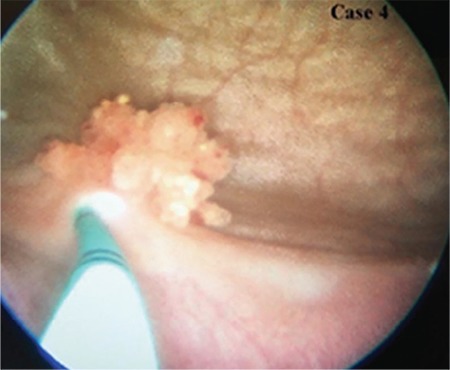
Perioperative cystoscopic view of the urinary bladder tumor, showing polypoidal luminal growth of patient's 4.

**Figure 3:**
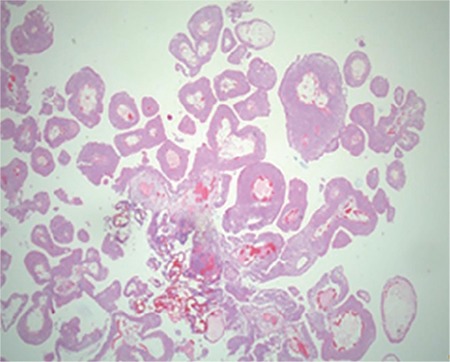
Histological outcomes of patient 4. Atypical urothelial cells and papillary structures were observed around fibrovascular cores, without lamina propria invasion.

Four patients aged <18 years diagnosed with UC and treated in our clinics between 2001 and 2015 were retrospectively evaluated. The demographic data of patients are summarized in Table 1. Although the four patients attended our clinic for different reasons, macro- or microscopic hematuria was a common finding. Smoking was detected as a risk factor for bladder tumor in only one of our patient (patient 2). In all the patients, a protruding lesion was detected in the bladder lumen on ultrasonography (USG) (Figure 1). All the patients, except patient 1, underwent complete resection under general anesthesia using 13- and 24-Fr resectoscopes, and were diagnosed with UC of the bladder (Figure 2). Patient 1 presented with different complaints, and underwent open surgical excision instead of endoscopic excision because of the physical conditions of our clinic at that time. None of the patients developed any complication during or after surgery. Urethral catheters were removed, and the patients were discharged on postoperative day 2. All the patients had the same pathologic diagnosis: low-grade, parent-teacher association, noninvasive transitional cellular carcinoma (TCC) (Figure 3). The patient follow-up protocol at our clinic includes USG examinations performed postoperatively at every 3 months for 6 months, every 6 months for 2 years, and at least every year for the subsequent 3 years. Furthermore, cystoscopy is also performed every year for postoperative 2 years. None of our patients experienced any recurrence or complication during the follow-up period, and no additional treatment was required after transurethral resection.

Informed consent was obtained from the parents of each child.

## DISCUSSION

UC of the bladder is extremely rare in children. Its incidence has been reported as 0.4% in individuals aged <20 years and 0.03% in those aged <16 years (1). In 2016, 5068 patients were admitted to our pediatric outpatient clinic, and a total of 420 surgeries were performed, excluding minor cases (e.g., circumcision). Between 2001-2015 only four cases of UC was detected. In our case series, the age range of the patients was 10-17 years, with a mean age of 13.7 years. According to the 1973 World Health Organization (WHO) classification, UC tumors in pediatric patients were histologically graded as well differentiated (grade 1), moderately differentiated (grade 2), and poorly differentiated (grade 3) TCCs. However, this classification has been updated by WHO/International Society of Urologic Pathologists consensus classification published in 2004 to papillary urothelial neoplasms of low malignant potential (PUNLMP), low-grade papillary UCs, and high-grade papillary UCs, respectively (Table 2) (2). Pathological evaluations, recurrence rates, and follow-up details of previously reported case series are summarized in Table 3. Recurrence rates of 2.6%-13% have been reported in patients aged <20 years with epithelial tumors (3); these rates were much lower than those for adult patients (40%-70%) (4). This difference may be attributed to the low malignant potential of these low-grade tumors. However, other studies have reported higher recurrence rates for pediatric patients still lower than adults (2,3). Paner et al. (5) reported recurrence and progression rates in patients with UC aged <20 years as 3.4% and 1.1%; respectively. All the patients in our study had noninvasive UC, and none developed any recurrent tumor during the follow-up period (mean duration, 4.8 years). Currently, there are no guidelines for the treatment and follow-up of pediatric UC cases. The management of pediatric UC is based on the experience obtained with adult patients. The main treatment modality includes transurethral resection (TUR) of the bladder tumor in most cases. Early single-dose intravesical chemotherapy is used postoperatively to reduce recurrence in adults, but there is insufficient knowledge about its indications and usage for the pediatric patient group. The effectiveness of intravesical immunotherapy, which is used in adult patients following TUR, has not been elucidated in children because of the rarity of cases. Radical cystectomy, partial cystectomy, and systemic chemotherapy may be reserved as treatment options for children with high-grade or muscle-invasive UCs. USG is the most commonly used modality for the postoperative follow-up of pediatric patients; it is noninvasive and highly sensitive (3). Although the sensitivity of computed tomography is similar to that of USG, it is not recommended for the follow-up of pediatric patients because of the risks of exposure to ionizing radiation and high cost involved (3). Urinary cytological screening can also be used as a noninvasive method for follow-up; however, it is often not helpful in practice, because UC tumors are low-grade, and the sensitivity of this screening method ranges between 6% and 38% (6). Cystoscopy is the gold standard for the postoperative follow-up of pediatric patients with UC, despite its disadvantages, such as the requirement for general anesthesia and possibility of developing urethral trauma. Two-thirds of recurrences may be asymptomatic and can only be diagnosed using cystoscopy (7). Postoperative follow-up of pediatric patients with UC is controversial, and urologists have developed a number of follow-up protocols based on the knowledge and experience gained from adult patient practice. Polat et al. (8) reported that USG alone is sufficient for follow-up, and that cystoscopy should only be used when a tumor is suspected in this age group which has low recurrence rate. Similarly, Lerena et al. (6) reported USG as a follow-up modality for pediatric patients with UC at 1 week, 3 months, and 6 months postoperatively and annually thereafter, recommending cystoscopy only for patients with tumor recurrence. As emphasized by these two reports, follow-up with USG alone seems to be sufficient because of the low recurrence rate of these noninvasive, low-grade tumors. Conversely, some authors have suggested that cystoscopy should be included in routine follow-up protocols. Ander et al. (9) proposed similar follow-ups for pediatric and adult patients. They monitored pediatric low-grade tumors (transient axonal glycoprotein 1 and PUNLMP) in the postoperative period with USG and cystoscopy at 3 and 9 months, followed by USG twice a year and cystoscopy once a year for the subsequent years. For high-grade tumors (T1G1), they preferred performing USG and cystoscopy at 3, 6, and 12 months postoperatively and cystoscopy twice a year in the following years. We followed our patients in the postoperative period with USG (at postoperative 3, 6, 12, 18, and 24 months) and cystoscopy (at postoperative years 1 and 2). Then, we continued the follow-up with yearly USG and urinalysis for at least 5 years. We believe that USG and cystoscopy are sufficient for the follow-up of these patients in experienced centers (10). The limitations of this study included its retrospective design and the limited number of cases.

In conclusion, pediatric UCs are rarely encountered low-grade, low-stage tumors, with a low recurrence rate after treatment. Because of the limited number of cases, there are no widely accepted treatment and follow-up protocols. Thus, larger patient series with multi-centered studies are needed to establish specific follow-up protocols for this age group.

## Figures and Tables

**Table 1:**
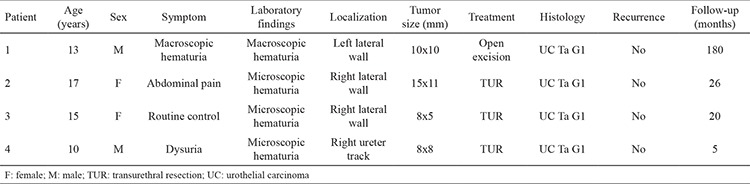
Patients’ demographic data can be used

**Table 2:**
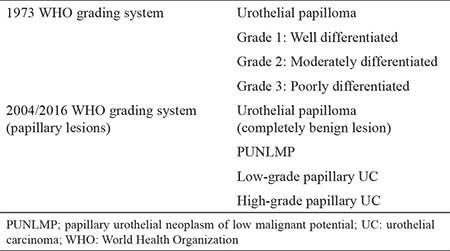
The 1973 World Health Organization and 2004 World Health Organization/International Society of Urological Pathology consensus classifications

**Table 3:**
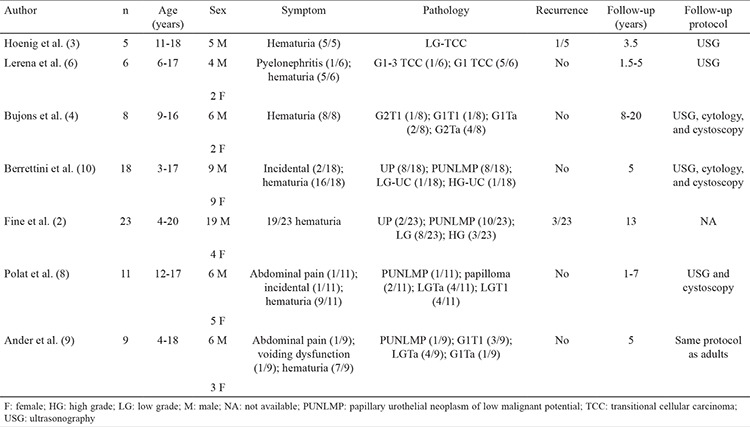
Previously published case series of pediatric urothelial carcinoma
